# People that Deliver Theory of Change for Building Human Resources for Supply Chain Management: Applications in sub-Saharan Africa and Southeast Asia

**DOI:** 10.9745/GHSP-D-23-00467

**Published:** 2025-05-09

**Authors:** Pamela Steele, Hilary Claire Frazer, Gashaw Mekonnen

**Affiliations:** a Pamela Steele Associates, Kisumu, Kenya.; b Mozen Consulting Ltd., London, United Kingdom.; c Management Sciences for Health, Washington, DC, USA.

## Abstract

The Theory of Change for Building Human Resources for Supply Chain Management (TOC) offers a practical framework outlining 4 interdependent pathways—staffing, skills, working conditions, and motivation—to manage the workforce quantity and capability necessary to operate health supply chains effectively. We conducted a desk review of project reports on applications of the TOC as a diagnostic and analytical framework for health supply chains in Cameroon, Ethiopia, Malawi, Rwanda, and the Philippines. We compared approaches to program development, project management, and implementation to reach conclusions and make recommendations based on experience in each country. The TOC can be applied in multiple country contexts, is useful in highlighting supply workforce challenges, and provides a framework that allows governments and technical partners to readdress them.

## Abstract (precis)

The Theory of Change for Building Human Resources for Supply Chain Management was applied in 5 countries to help highlight existing supply chain workforce challenges and provide a framework for addressing them.

## Teaser

**Key Findings:**

- The Theory of Change for Building Human Resources for Supply Chain Management (TOC) was applied in Cameroon, Ethiopia, Malawi, Rwanda, and the Philippines to identify gaps and prioritize workforce interventions needed to strengthen the health supply chains (SCs) in these countries.
- The SC projects and interpretations of the TOC varied according to country context, donor priorities, and other external factors.
- Applying the TOC resulted in some favorable outcomes to help address improvements in the SC workforce.

**Key Implications:**

- The TOC enables SC practitioners to capture and understand the pathway of change that connects interventions and investment in human resources to health SC performance improvements and improved health outcomes.
- This tool allows governments and technical partners to articulate the complexities that govern human resources in health SCs and identify how interventions can improve the staffing, skills, motivation, and working conditions of this workforce.

## INTRODUCTION

Human resources (HR) for health has recently received more attention globally, and recognition of the importance of HR for supply chain management (SCM) has become more widespread, especially since the COVID-19 pandemic, highlighting the value of the workforce, its abilities and motivations, and its potential to strengthen supply chains (SCs).^1^ Although SC labor shortages persist in many contexts, particularly in low- and middle-income countries (LMICs), little research examines HR in health SCs.^2^ Data on the SC workforce frequently has gaps, especially at the administrative level, which causes capacity requirements to remain undiscovered.^3^ In addition, SC workforces often lack the technical skills and managerial competencies to optimize performance, with insufficient numbers of competent staff potentially causing breakdowns in SC systems.^4^

HR challenges for LMICs are often compounded by poverty; a high burden of disease; insufficient facilities for growing and migrating populations; inadequate resources, especially for primary care in remote areas; and humanitarian response to natural and other emergencies.^5^

In East Africa, issues affecting HR in health have been particularly significant because of frequent acute labor shortages in SCs. The capacity of HR for health has been affected by outdated education and traditional management practices. Additionally, data on SC employees frequently have gaps and discrepancies, especially at the administrative level, with health care workers left uncounted because information systems in low-income countries are weak.^6^ In this article, we define HR as a “workforce” to denote the group of people who work in a company, industry, or country, which is distinct from the HR directorate. Traditionally, HR directorates in the African public sector have not been featured in strategic decision-making.

By contrast, countries in the Southeast Asia region committed to a decade of strengthening HR for health from 2015 to 2024. In the Philippines, studies have shown a promising upward trend in hiring efforts to meet the country’s health care demands, with a year-on-year increase of 26% in hiring in the sector between 2022 and 2023.^7^

Organizations have had to respond more effectively to external disrupters, including political and economic developments. Other outside influences, such as social media, have changed workforce expectations. The COVID-19 pandemic has had a lasting impact on working practices and employment relationships.^8^

Subsequently, recognition is building that business managers need to know more about their workforces to perform effectively. However, it is also imperative that HR professionals understand their business units. Integration of these perspectives can enable organizations to compete on a global scale, attract foreign investment, and promote cross-border collaboration.^9^ There is an increasing need for HR managers to shift their focus toward activities that add more value to their organizations, such as developing a talented and motivated workforce to improve public service delivery. Efforts to improve SCs must acknowledge the value of its people, their abilities and motivations, how they are managed, and their potential for personal and professional growth. Empirical research has shown that the strategic management of people’s knowledge, skills, and talents favors SCM success, organizational performance, and customer satisfaction.^10^

Consensus is forming that health outcomes in LMICs will only continue to improve if the capacity and skills of the health SC workforce are developed. As such, there has been an increased focus on systematic approaches to HR capacity development and the professionalization of SCM cadres, particularly in LMICs. Collaborations with the Medicines, Technologies, and Pharmaceutical Services Program, the U.S. Agency for International Development’s (USAID) Global Health Supply Chain Procurement and Supply Management (GHSC-PSM) project, and the Human Resources for Health in 2030 program recognize this and support governments in assessing needs and developing strategies to manage the quantity, type, and capacity of HR required to health SCs.^4^

The Theory of Change for Building Human Resources for Supply Chain Management (TOC) was developed to describe the impact of interventions and investments in HR for SCM that aim to improve SC performance. ^4^ It offers a practical framework that can be used to prioritize the workforce interventions required to strengthen health SCs.

The TOC offers a practical framework that can be used to prioritize the workforce interventions required to strengthen health SCs.

Through 5 use cases in the public sector, we show how this TOC has been applied as a diagnostic and analytical framework in Cameroon, Ethiopia, Malawi, Rwanda, and the Philippines. Findings from a desk review of project reports compare approaches to program development, project management, and implementation to reach conclusions and make recommendations based on experience in each country.

## PURPOSE AND APPLICATIONS OF THE THEORY OF CHANGE

The TOC comprises 2 parts: the diagram (Figure and Supplement 1) and the narrative (Table). The Figure shows the following 4 interdependent pathways and their corresponding preconditions to improve HR in health SCs effectively.

- Staffing: All critical positions and/or competencies are filled.
- Skills: Workers apply their skills as appropriate at every level of the SC.
- Working conditions: Working conditions support performance.
- Motivation: SC workers are motivated to do their jobs.

**FIGURE:**
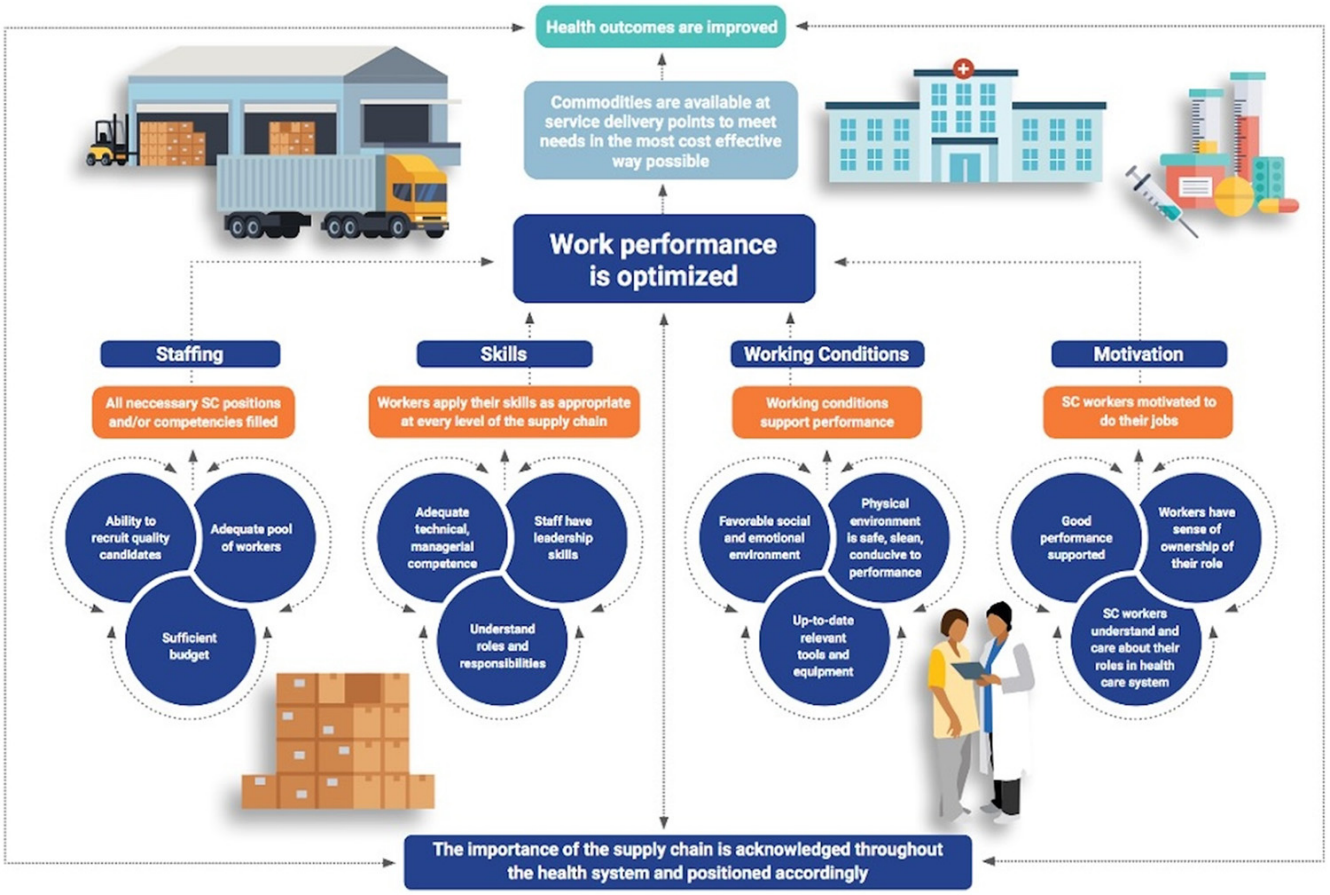
Simplified Version of the Human Resources for Supply Chain Management Theory of Change Model

**TABLE.** Nine Critical Assumptions That Underlie the Human Resources for SCM Theory of Change Diagram

**Assumption**	**Description**
Optimizing SCM work performance will lead to improved commodity availability and improve health outcomes.	People power SCs. An appropriately organized and adequately staffed workforce with the required, specialized technical skill sets are needed to make SCs function effectively.
Acknowledging the importance of SC enhances efforts to optimize workforce performance.	Recognizing the importance and complexity of SCM is essential to building national health systems where well-managed SCs ensure the availability of critical commodities and allow for health care delivery.
Adopting a crosscutting approach will lead to greater results.	Interventions that work across various levels of the organization and the SC are more likely to have an effective and sustained impact.
Cadres that perform SCM vary between different country contexts.	The TOC can be applied equally to SCs managed by various cadres.
The public sector SC can leverage private sector best practices to create change.	The public sector can apply private-sector solutions to HR issues to reach the desired outcomes. Lessons learned from applying private-sector tactics to public-sector SCs have shown that the private sector can play an important role in improving health SCs.
Public health SCs are often neither entirely embedded in the public sector nor in the private sector.	SCs may intersect both public and private sectors through use of contracting and outsourcing, parastatal entities, and/or other hybrid approaches. The pathways and outcomes within the TOC can be applied to any and all of these scenarios.
Well-functioning HR management systems—with effective policies and well-defined processes—enable optimized work performance.	Institutional-level outcomes and interventions, such as introducing policies and creating and implementing effective HR management processes, are foundational steps toward building an efficient workforce.
Strengthening the role of SC leadership and supervisors will lead to optimized work performance.	Empowering individual-level supervisors and SC leaders will bolster the overall performance of the SC workforce.
Cultivating workers’ motivation and skills improves work performance.	Creating opportunities to improve individual-level competencies and motivation will lead to improved work performance.

Abbreviations: HR, human resources; SC, supply chain; SCM, supply chain management; TOC, theory of change.

Source: Brown et al.^4^

The narrative describes the components and 9 critical assumptions that support interventions around HR for SCM (Table).

The TOC was developed by People that Deliver (PtD), a coalition established in 2011 as a global partnership of organizations focusing on the professionalization of SC personnel by advocating for a systematic approach to HR for SCM at the global and local levels. PtD advocates at the international and national levels for interventions that improve the demand and supply of qualified health SC professionals in organizations, which, in turn, strengthens the individual practitioners within those organizations. Since the establishment of PtD, there has been an increased focus on systematic approaches to HR capacity development and the professionalization of SCM cadres, particularly in LMICs. One of PtD’s founding principles was to recognize the importance and complexity of SCM. Doing so is essential to building national health systems in which well-managed SCs provide critical commodities and allow for health care delivery.

A TOC explains how a given intervention or set of interventions is expected to lead to specific development change, drawing on a causal analysis based on available evidence.^11^ It follows a crosscutting approach and provides a unifying framework to define a need or goals and a realistic scope; identify assumptions; consider enablers to address what works, how, where, for whom, and at what cost; plan activities or interventions (inputs and outputs); plan for the changes expected (outcomes); and identify the differences these will make (impact).^12^^,^^13^ It can also be used as a diagnostic tool by concisely capturing complexity, for example, identifying what is necessary to manage the quantity, type, and capacity needed to optimize the workforce and improve SC performance. A TOC can also help define success.^14^

## THEORY OF CHANGE IMPLEMENTATION USE CASES

In 5 countries—Cameroon, Ethiopia, Malawi, Rwanda, and the Philippines—the TOC was used as a diagnostic and analytical framework for program development, project management, and delivery. These initiatives, which took place over a 4-year period from Quarter 3 2018 to Quarter 4 2022, focused on functional SCM analysis for national health care systems and workforce assessment. It is important to consider the context in which projects were implemented and variables, such as structural issues and geopolitical challenges, civil unrest, and the COVID-19 pandemic (Supplement 2 provides details on each country). Specific themes for each initiative varied according to country context, donor priorities, and/or Ministry of Health (MOH) requirements. As the TOC is currently open source, the approaches differed rather than follow a standard set of procedures.

A universal health care model has been introduced in Rwanda, Malawi, and, recently, in the Philippines and is intended as a pillar for health sector transformation in Ethiopia. Public and private health care facilities are usually found in the capital, second, or third cities, with out-of-pocket (OOP) expenditure necessary for advanced or specialist treatment. Services can be limited and underresourced in remote areas.

The research for this article centered on a desk review of the reports for each initiative. The findings have been mapped to relevant critical assumptions and each of the 4 TOC pathways (Figure and Supplement 1).

### Cameroon

#### Overview

Given a lower middle-income classification by the World Bank in 2022, Cameroon’s health care system combines private and public institutions with companies offering multiple health care services.^15^^,^^16^ Under government-funded schemes, most of the country’s citizens receive free health care, which is co-funded by public and private enterprises, religious missions, foreign aid donors, and nongovernmental organizations (NGOs). Most of the 154 operational hospitals in the country are in major cities. With health spending at US$58.00 per capita and OOP spending at 68.3%,^17^ direct payments for on-demand health services depend on the person’s own financial means.^18^

The SCM initiative, which was conducted from July to September 2019, aimed to revamp primary health care and build a strong SC that ensures the availability and accessibility of essential health commodities. Funding was provided by the USAID GHSC-PSM project in partnership with the Directorate of Pharmacy, Medicine, and Laboratory. The initiative operated on 2 assumptions: (1) strengthening the role of SC leadership and supervisors will lead to optimized work performance, and (2) cultivating workers’ motivation and skills improves work performance.

In Cameroon, the SCM initiative aimed to build a strong SC to ensure the availability and accessibility of essential health commodities.

To ascertain the status of preconditions along the 4 pathways, the TOC HR Rapid Assessment tool was applied across public and private sector health sector units and departments by holding interviews, focus groups, and observations; gathering and analyzing workforce data to generate information about the status of HR in the health SC; conducting a workshop to validate findings and develop recommendations; documenting and mapping SC workforce designations, organizations/departments, SC functions, and levels in the public sector; and evaluating SC maturity and identifying progress in the public sector.

#### Examples of Requirements and Recommendations by Pathway

Regarding staffing, the following were needed: (1) an appropriate recruitment system; (2) adoption of a more equitable pay scale aligned with qualifications and competencies, supported by periodic salary market analyses; and (3) alignment of continuing professional development/education opportunities with career progression.

For skills, processes to create, adapt, and update job descriptions were needed, as well as review and approval; all staff needed a staff development plan; and senior personnel needed to review the organogram to ensure authority and accountability.

Efforts were needed to define and create a safe and conducive work environment with new and improved staff orientation and onboarding processes, comprehensive staff and supervisor training on harassment and discrimination protocols, and solution-focused coaching for leadership.

Regarding motivation, supervisors needed training to provide feedback on poor performance and take disciplinary action; managers needed training to adopt management styles that enabled workers to make decisions and take ownership of their tasks and success; and new communication channels were required to inform staff about their contribution to the health care system.

#### Outcomes and Impact

At first, the results of the HR assessment were supported by an MOH technical working group, and recommendations were received positively by MOH representatives. However, senior MOH management and relevant stakeholders’ buy-in had to be secured and continued over a prolonged period. Subsequently, key stakeholders left positions at the funder, and the country’s COVID-19 pandemic response took priority, so initiative implementation was disrupted. Nonetheless, an indirect outcome was the creation of the International Association of Public Health Logisticians Cameroon chapter, which enabled practitioners to share knowledge and information about managing critical supplies in clinics, hospitals, and across Cameroon.

### Ethiopia

#### Overview

Classified as a low-income country,^15^ the Ethiopian government set the target of attaining middle-income status by 2030–2035, with universal health coverage a key pillar of social and economic development. Consequently, nationwide transformation programs are underway. The MOH has embarked on the Health Sector Transformation Plan and Pharmaceutical Sector Transformation Plan II 2020/21–2029/30. The Ethiopian Pharmaceutical Supply Service (EPSS, formerly the Ethiopian Pharmaceutical Supply Agency, [EPSA]) is also expected to play a key role in repositioning the country as a regional pharmaceutical distribution hub for East Africa.

Ethiopia has a three-tier health care system with health spending at US$29 per capita.^17^ The government provides free health care, while private hospitals can provide treatment for major health issues. The availability of equipment and qualified staff is limited, especially in remote regions.^19^ The situation has recently been complicated by civil war.

This SCM initiative, funded by the Gates Foundation, aimed to improve EPSA’s SC operations based on the Gates Foundation’s Supply Chain Maturity Model by adopting the TOC as a project management framework to develop SC and HR directorates’ technical and behavioral capabilities. This project was part of Admas, a multiyear program aimed at enabling national health care transformation for Ethiopia and realizing organizational change for EPSA by following the strategic direction set out in the transformational plans and delivered by a partner consortium led by Pamela Steele and Associates.^20^ Three critical assumptions were made: (1) the public sector SC can leverage private sector best practices to create change, (2) public health SCs are often neither entirely embedded in the public sector nor the private sector, and (3) well-functioning HR management systems—with effective policies and well-defined processes—enable optimized work performance.

The program ran from 2018 to 2021. During this time, EPSA changed its director general, experienced organizational restructuring, and prepared for a 5-year SAP Enterprise Resource Planning systems implementation. Ethiopia also went through a period of national unrest that affected the delivery of health care products and services across much of the country.

#### Examples of Requirements and Recommendations by Pathway

For staffing, a proclamation was developed that would have given EPSA full control of the hiring process and have allowed more power to recruit and dismiss staff based on their skills and experience. This proclamation reached the Prime Minister’s office but was not approved during the project. Standards were defined for HR master data, a comprehensive audit was conducted, and data collection was centralized in preparation for an HR information systems implementation.

For skills, a tailored competency framework was created, outlining the skills, knowledge, and abilities needed for different roles in the organization. HR strategic plans were developed and updated with modern concepts. Workshops were hosted to establish standard operating procedures for consistent SC and HR professional practices. Temporary training centers at EPSA’s headquarters were established, as were branches with formal classes, on-the-job training, and mentoring to support study cohorts with SC professional certification.

Staff surveys revealed low commitment among staff and dissatisfaction with working conditions and safety practices. A culture diagnostic was run, and a strategy was developed that included new employee induction and culture ambassador programs, a code of conduct, and a gender audit in collaboration with the Women’s Strategic Development Centre. A compensation review and benchmarking study were conducted to compare the pay range in the SC industry. These findings were used to recommend a new grading system and compensation structure that would be fair and competitive.

For motivation, performance management best practices were reviewed and recommended, and technical committees were supported through performance management processes. It was advised that the organization transition from paper-based to electronic appraisal systems.

#### Outcomes and Impact

EPSA achieved Supply Chain Maturity Model silver status based on self-assessment and, as advised by Pamela Steele and Associates, initiated comprehensive workforce data collection. They were also readied for subsequent interventions, such as PtD’s STEP 2.0 leadership development program.

The EPSA achieved Supply Chain Maturity Model silver status based on self-assessment.

### Malawi

#### Overview

Malawi is classified as a low-income country.^15^ The public sector provides 52% of the health services, and patient care is funded in part by the National Health Insurance Scheme; private and nonprofit facilities operate alongside. Private medical insurance is negligible in financing health care, with OOP spending reported at 10.3%.^17^ The affordability of medical costs at private/Christian Health Association of Malawi facilities and transport costs remain the main access barriers in remote and rural areas.^21^

This SCM initiative, funded by The Global Fund, aimed to identify and address strengths and weaknesses of key aspects of workforce management and development for health SCM. This project focused on 3 target groups working across the public and private sectors: (1) the Central Medical Stores Trust (CMST), responsible for procurement, warehousing, sales, and distribution of medicines and medical supplies; (2) the Program Implementation Unit of The Global Fund, responsible for all programmatic reporting of pooled funds; (3) Cargo Management Logistics and Bolloré Transport & Logistics, which are private companies specializing in warehouse services and distribution, freight forwarding, and logistics.

The project, which was conducted in Quarter 3 of 2019, also applied the rapid diagnostic tool as a basis for focus group discussions. The PtD Health SC competency compendium was also used as the basis for a skills assessment. This followed an earlier review in 2018, which identified challenges with civil servant organizational culture. Two critical assumptions were that public health SCs are often neither entirely embedded in the public sector nor the private sector, and cultivating workers’ motivation and skills improves work performance.

#### Examples of Requirements and Recommendations by Pathway

For staffing, the efficiency of recruitment processes was reviewed and measured according to the number of vacancies and time taken to hire personnel, which varied depending on duration and transparency. Competency-based recruitment was recommended.

For skills, job descriptions needed updating to meet industry standards.

For working conditions, the development of a staff retention scheme was suggested.

For motivation, a training needs analysis was prepared with recommendations for developing and implementing learning programs.

#### Outcomes and Impact

The recommendations provided from the assessment were incorporated into the CMST’s business plan and corporate strategy, enabling them to track overall organizational development using the TOC rapid diagnostic tool and competency compendium regularly.

Following the HR assessment in Malawi, CMST attained a budgeted training plan, planned for HR policy revisions, and requested reforms allowing a salary scheme, career planning, and promotion systems to be managed organically without needing civil service involvement and approval.

### Rwanda

#### Overview

Although still classified as a low-income country,^15^ Rwanda has been rebuilding its economy. Rwanda follows a universal health care model through the mutuelles de santé, a mandatory community-based health insurance scheme. Although considered a high-quality health system, hospitals may lack basic equipment, electricity, and running water. It is ranked 110 of 148 countries for health survival.^22^ Half the actual cost of care per citizen comes from government sources and the remainder from international donors. Private health care has been growing steadily since 1994, and more than 50% of private health care facilities operate in or near Kigali. In 2020, government health spending was 40.0% of total health spending, and OOP spending was at 10.3%.^23^

The SCM initiative, conducted in Quarter 3 of 2018, aimed to assess the Rwandan HR system to identify the TOC outcomes in place and reveal which needed to be strengthened through targeted interventions to support the integrated health SC. The USAID GHSC-PSM project supported the MOH in Rwanda to document the state of HR capacity and management of the Rwandan public health SC, document the professionalization efforts of personnel working across Rwanda’s public health SCs, and develop recommendations and suggest a strategy and implementation roadmap for building the organizational and individual capacity. The critical assumption was that well-functioning HR management systems with effective policies and well-defined processes enable optimized work performance.

#### Examples of Requirements and Recommendations by Pathway

For staffing, it was suggested that SCM content in university curricula be developed to deepen the candidate pool. Job descriptions for SCM functions were to be developed to a defined MOH standard. Other recommendations included forecasting SCM positions in the MOH staffing structure, advocating SCM HR budget needs and allocating accordingly, and establishing a recognized SC cadre with ongoing educational opportunities.

For skills, a competency framework for SCM roles and responsibilities at all levels was to be established, putting in place staff development plans and implementing education and training interventions with corresponding SCM qualifications.

For working conditions, the required characteristics for a safe and conducive environment with a checklist for confirmation were to be developed, as were lists of required tools and equipment for each level. In addition, training materials needed to be developed and shared with all staff.

For motivation, training supervisors needed to provide feedback on poor performance and take disciplinary action. Improving orientation and onboarding processes was advised with other communication channels to inform staff about their contribution to the health care system.

#### Outcomes and Impact

Applying a structured framework aided in the design and development of interventions. Consensus-building was improved during the initial workshop and data collection. A comprehensive, systematic process was applied to assess strengths and weaknesses and identify gaps. Indicators were provided to monitor the performance of HR systems. Stakeholder engagement and buy-in were enhanced by providing insight into the methodology and visibility of the criteria being assessed, showing how recommended interventions could work synergistically with existing processes to improve implementation, enabling a shared understanding of the complexities of HR in health SCs and navigating to create change, and clearly depicting outcomes that needed to be in place within the HR management system. Consequently, the Rwanda MOH prioritized future HR for SCM investments to improve the availability of skilled cadres required for the ongoing effective management of health SCs.

The Rwanda MOH prioritized future HR for SCM investments to improve the availability of skilled cadres required for the ongoing effective management of health SCs.

### Philippines

#### Overview

The health care system in the Philippines combines public and private options. Since universal health care was established in 2019, all Filipino citizens have been entitled to free health care under the Philippine Health Insurance Corporation, which is partly funded by government subsidies and company payroll deductions. Government spending as a percentage of health care expenditure increased from 33.1% in 2005 to 44.6% in 2020.^17^ State-subsidized public health care is comprehensive. However, quality can vary between facilities; health care from private hospitals is more widely available in major cities than in rural areas.^24^

The initiative, funded by USAID’s Medicines, Technologies and Pharmaceutical Systems program in collaboration with the Department of Health (DOH), was intended to inform the acquisition, retention, and capacity-building of the Philippines’ SCM workforce, systematically address gaps, and resolve shortages at the central, regional, provincial, and facility levels. The critical assumptions were optimizing SCM work performance will lead to improved commodity availability and improved health outcomes, adopting a crosscutting approach will lead to greater results, and cadres that perform SCM vary between different country contexts. Conducted from 2020 to 2022, the initiative focused on defining the functions, roles, and responsibilities within the policies and guidelines in place to deliver universal health care, based on the DOH’s mandate, through the following activities.

- Conduct a functional analysis of the SCM workforce needs using the Supply Chain Operations Reference model, the cross-industry, standard diagnostic tool for SCM that was developed and endorsed by the Supply Chain Council, now a part of the Association for Supply Chain Management.
- Understand the existing skills, competencies, and behaviors through key informant interviews, questionnaires, and facilitated group discussions based on the PtD competency compendium.
- Assess the existing positions, numbers, job descriptions, and skillsets of the existing SCM workforce to determine similarities, gaps, and overlaps compared with the 6 PtD domains.
- Review the organization design to recommend required positions, numbers, and distribution.
- Consider the requirements and prepare the rationale of the DOH to develop SCM workforce plans with expected roles and suggested roadmaps.
- Present proposals to the Department of Budget and Management justifying the need to increase and train for regular positions.
- Define measures to hire the right people in the right quantity for the right position and strengthen and develop the capacity of SCM personnel needed to ensure the skills are available in the future.

#### Examples of Requirements and Recommendations by Pathway

Although the project scope focused on staffing and skills by adopting a crosscutting approach, interventions were also attributed to the working conditions and motivation pathways.

For staff, a review of recruitment and hiring practices found that 28% of warehouse and distribution staff reportedly had short contracts (for 6 months), and some were hired with donor funds. During the project, the SC management team received approval to hire 69 additional staff in 2019–2020, although in an ad hoc manner.

For skills, staff were categorized into 4 groups according to 4 SCM competency domains. Skillsets, behaviors, and associated tasks were aligned to clarify job responsibilities and key tasks, compared with the PtD domains. The requirements for approved positions and capacity gaps were identified for existing staff at the individual, organizational, and DOH/environmental levels.

For working conditions, an organizational structure for SCM was recommended that determined end-to-end SC functional areas and all entities in and outside of the DOH, with critical roles and responsibilities to fulfill. A workforce development plan was designed and established as the foundation of the DOH’s ongoing commitment to its workforce. Goals were set to ensure that an adequate and well-supported SC workforce was deployed appropriately and provided with the requisite equipment and working environment and to develop a shared culture.

For motivation, it was noted that SC staff were not part of the HR for health workforce projected in the master plan 2020–2040, which was already underway. Employing staff on short contracts may not have been sustainable beyond project cycles, but this created uncertainties, which may have weakened motivation.

Developing a competency framework would standardize assessment, feedback, and communication about performance. This would manage workforce training and succession planning objectively, reward good performance, and ensure productivity and retention of high-quality staff.

#### Outcomes and Impact

Quality improvement and staff retention were included in a comprehensive strategy that incorporated training and capacity development of the SC workforce through the identification of gaps in skills and knowledge. More permanent SC staff positions were being approved to select and hire SC staff appropriately. Nonetheless, the unavailability of skilled and trained public health SC staff, reliance on short-term contracts and secondments, and attrition remained challenges.

In the Philippines, quality improvement and staff retention were included in a comprehensive strategy that incorporated training and capacity development of the SC workforce.

## DISCUSSION

We have shown that the TOC could be applied in multiple country contexts to highlight health SC workforce challenges and offer a framework that allows governments and technical partners to address them.

### Inconsistent Application of the Theory of Change

Currently, the TOC is “open source” to allow the TOC’s widespread availability and encourage uptake. This, though, can present challenges.^25^ Rather than adhere to the methodology and sequencing described in the TOC, the approaches in the 5 use cases varied. For example, the TOC was used as an assessment tool in Cameroon, a project management framework in Ethiopia, and in the development of rapid action plans in Malawi.

Although allowing flexibility, this may leave room for confused relationships between the TOC and project log frames among initiatives within an extended program and/or between otherwise comparable projects across locations.^25^ Nonetheless, the TOC can be used as a learning aid, helping to identify when it becomes apparent that an intervention cannot meet its goals because assumptions are not being met.^12^

### Political Commitment Varied

The willingness of governments and institutions to adopt policy changes and revise legal mandates and their preparedness to implement a new and potentially empowering organizational design if policy changes are granted may also vary. The stage of organizational development may depend on limitations in the skills and commitment necessary for enacting a TOC at various levels,^25^ as may have been the case when the Ethiopian MOH delayed the proclamation approval, which would have allowed EPSA more flexibility to manage its own workforce.

### High Turnover Affected the Effectiveness of Interventions

Public health SC institutions struggle with staff retention, which is often influenced by low wages and poor working conditions. Constant recruitment and rapid training cycles may affect all pathways as qualified staff members leave for better opportunities in NGOs, United Nations agencies, or the private sector. Consequently, the organization would be constantly hiring and onboarding. At the same time, remaining colleagues would have an increased workload, lack professional development, and/or suffer uncomfortable and unsafe working conditions, which would also affect staff motivation.

There were risks associated with hiring 28% of warehousing and distribution staff on short-term contracts in the Philippines. This can directly affect levels of workforce participation and the capacity to deliver essential health services to the communities and users they cater to.^12^

## RECOMMENDATIONS

When applying the TOC to identify and plan interventions to address SC workforce challenges, we make the following recommendations.

### Consider the Specific Context

External factors (e.g., colonial history, political change, and varying levels of economic development) can all affect strategic ambitions for health care and lead to different interpretations of the TOC depending on the country context.^25^ For example, President Ferdinand “Bong Bong” Marcos Jr. had recently been elected as President of the Philippines at the time of the initiative, national unrest had previously been a feature in Rwanda, and Ethiopia had experienced weather-related events, such as drought, flooding, and typhoons.

### Build a Broad Coalition of Support

Alongside the implementation of a TOC, NGOs, United Nations agencies, and the private sector should support public health SC institutions by offering resources, sharing best practices, or partnering in initiatives. This helps to build a common team and foster a shared stakeholder understanding of what is to be achieved, thus contributing to the sustainability of outcomes.^26^ However, each stakeholder may have a different interpretation and may not share the same priorities, leading to incoherence in relationships among the constituent concepts.^25^ For example, recommendations were made for an appropriate recruitment system and equitable pay scale to improve the staffing situation in Cameroon. Despite initial support from MOH representatives, key stakeholders left, and then the focus shifted to the COVID-19 pandemic, which delayed decision-making and implementation. Also, it was recognized that there was a need for a defined MOH standard to develop SCM job descriptions in Rwanda to improve the availability of skilled cadres and to foster a safe and conducive environment because terms of employment and working conditions varied due to varying levels of importance attached to investments in HR for SCM resourcing.

### Identify Assumptions and Determine Outcomes

Identifying assumptions is important to showing the steps between project activities, delivering program goals, and realizing longer-term impact.^12^ Identifying causal links between interventions and determining the relationship with outcomes during ongoing and/or unexpected change, as with projects delivered during the COVID-19 pandemic, can be difficult.

## CONCLUSION

The TOC offers a practical framework, identifying 9 critical assumptions and outlining 4 pathways to manage the workforce quantity and capability necessary to operate health SCs effectively. These pathways are interdependent, so advancements in one area may precipitate improvements in others.

Future research could include a lateral study to compare how the TOC has been used in a wider range of countries and could facilitate cross-regional experience transfer through a comparison of use cases. A longitudinal study could follow up and track the progress of expected outcomes over time. Independent evaluators could assess the effectiveness of these changes, periodically review the implementation of the TOC approach, and measure the impact of new policies or legal mandates. This could provide valuable insights for continuous improvement.

## Supplementary Material

GHSP-D-23-00467-Steele-Supplements.pdf

## Notes

**Peer Reviewed**
