# Supplementary material for: People that Deliver Theory of Change for Building Human Resources for Supply Chain Management: Applications in sub-Saharan Africa and Southeast Asia
Source: Glob Health Sci Pract. 2025 May 9;13(Suppl 1):e2300467. doi: 10.9745/GHSP-D-23-00467 (PMC12063751; doi:10.9745/GHSP-D-23-00467)
Supplement: GHSP-D-23-00467-Steele-Supplements.pdf [file GHSP-D-23-00467-Steele-Supplements.pdf]

Supplement to: Steele P, Frazer HC, Mekonnen G. People that Deliver theory of change for building human resources for supply chain management: applications in sub-Saharan Africa and Southeast Asia. *Glob Health Sci Pract*. 2025;13(Suppl 1):e2300467. https://doi.org/10.9745/GHSP-D-23-00467

SUPPLEMENT 1. Overview of the 4 pathways, pre-conditions and key components taken from the Theory of Change for Building Human Resources for Supply Chain Management

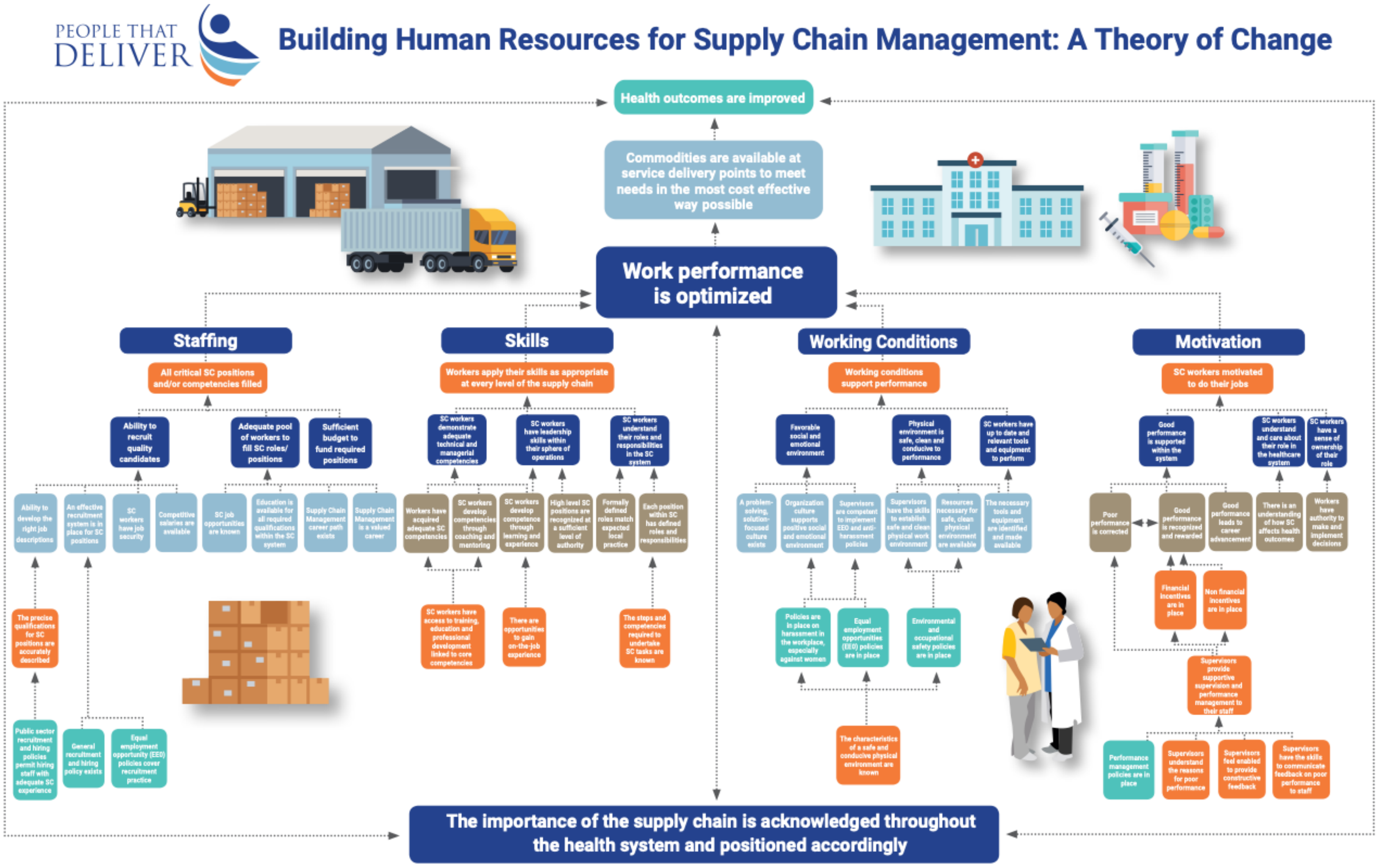

Supplement to: Steele P, Frazer HC, Mekonnen G. People that Deliver theory of change for building human resources for supply chain management: applications in sub-Saharan Africa and Southeast Asia. *Glob Health Sci Pract*. 2025;13(Suppl 1):e2300467. https://doi.org/10.9745/GHSP-D-23-00467

SUPPLEMENT 2. HEALTHCARE PROFILES BY COUNTRY

CAMEROON

Cameroon's healthcare system comprises a combination of entities, including private and public institutions, as well as companies that offer multiple healthcare services. Under government-funded schemes, most of the country's citizens receive free healthcare, which is funded by the Cameroon government, public and private enterprises, religious missions, foreign aid donors, and NGOs. Cameroon's public healthcare system provides inexpensive healthcare services and medicines to residents, and it works on three levels: operational, technical, and strategic. Most of the 154 operational hospitals in the country are located in major cities, such as Douala and Yaounde, and a few hospitals have medical professionals who are highly knowledgeable in global healthcare ethics and practices.

On the other hand, there are more than 50 private hospitals in Cameroon that supply modern equipment and premium private healthcare services. There are three main types of health insurance schemes in Cameroon: National Health insurance (NHI), community-based health insurance (CBHI), and private health insurance. However, direct payment for on-demand health services will be a factor of inequality to the extent that it depends on the patient's own financial means.

In summary, the healthcare options in Cameroon include:

- Public healthcare system that provides inexpensive healthcare services and medicines to residents.
- Private healthcare system that supplies modern equipment and premium healthcare services.
- National Health insurance (NHI), community-based health insurance (CBHI), and private health insurance schemes.

References

Expat Financial, Learn about the Cameroon healthcare system for expats. Accessed 12 June 2023. https://expatfinancial.com/healthcare-information-by-region/african-healthcare-system/cameroon-healthcare-system/

Tandi TE, Cho Y, Akam AJ, Afoh CO, Ryu SH, Choi MS, Kim K, Choi JW. Cameroon public health sector: shortage and inequalities in geographic distribution of health personnel. Int J Equity Health. 2015 May 12;14:43. doi: 10.1186/s12939-015-0172-0. PMID: 25962781; PMCID: PMC4440287.Accessed August 2023. https://www.ncbi.nlm.nih.gov/pmc/articles/PMC4440287/

On policy (2023) Private Health Insurance Service Providers in Cameroon: Current Status and Organizational Framework. Accessed August 20 2023) https://onpolicy.org/private-health-insurance-service-providers-in-cameroon-current-status-and-organizational-framework/

World bank (2013) Better Access to Health Care for all Cameroonians. Accessed September 2023. https://www.worldbank.org/en/country/cameroon/publication/better-health-care-access-for-all-cameroonians
https://www.usc.gal/economet/reviews/ijaeqs323.pdf

ETHIOPIA

Ethiopia has a three-tier healthcare system with primary, secondary, and tertiary levels of care. The Ethiopian government provides free healthcare to its citizens, but the quality of care is inadequate, and the existing hospitals lack adequate equipment and efficient human resources. Most hospitals are located in the capital city of Addis Ababa, and hospitals with full-time physicians are only found in major cities. In remote regions of the country, the quality of healthcare systems and infrastructure is poor. Private hospitals provide better care for major health issues, but they are expensive and not linked to any insurance system. People who need to be evacuated to a different country for medical treatment end up paying as much as USD$ 100,000.

References

Columbia (2023) Ethiopia Summary. Accessed September 21 2023. https://www.publichealth.columbia.edu/research/others/comparative-health-policy-library/ethiopia-summary

Pacific Prime. Ethiopia Health Insurance. Accessed September 10 2023. https://www.pacificprime.com/country/africa/ethiopia-health-insurance-pacific-prime-international/

Merga, B.T., Balis, B., Bekele, H. et al. Health insurance coverage in Ethiopia: financial protection in the Era of sustainable development goals (SDGs). Health Econ Rev 12, 43 (2022). https://doi.org/10.1186/s13561-022-00389-5. Accessed September 11 2023. https://healtheconomicsreview.biomedcentral.com/articles/10.1186/s13561-022-00389-5

MALAWI

Health services in Malawi are provided by the public, private for profit (PFP), and private not for profit (PNFP) sectors. The public sector provides the majority (52%) of the health services, with four levels of care: community, primary, secondary, and tertiary. The MoH, district, town and city councils, Ministry of Defence, and Ministry of Internal Affairs and Public Security provide public services in collaboration with the MoH. The National Health Insurance Scheme (NHIS) provides public healthcare for expats and locals. The private sector provides locally compliant international medical insurance plans and quotes. Private medical insurance plays a negligible role in financing healthcare in Malawi, and pooling is significantly fragmented with limited redistributive capacity. Over 60% of all health services are provided in public hospitals and health centres, 37% by the private not-for-profit Christian Health Association of Malawi (CHAM), and the rest by individual private-for-profit health practitioners. Affordability of medical costs at private/CHAM facilities and transport costs remain the main access barriers to seeking health care in rural Malawi.

References

Makwero MT. Delivery of primary health care in Malawi. Afr J Prim Health Care Fam Med. 2018 Jun 21;10(1):e1-e3. doi: 10.4102/phcfm.v10i1.1799. PMID: 29943590; PMCID: PMC6018651.Accessed 9 September 2023. https://www.ncbi.nlm.nih.gov/pmc/articles/PMC6018651/

Mchenga M, Manthalu G, Chingwanda A, Chirwa E. Developing Malawi's Universal Health Coverage Index. Front Health Serv. 2022 Feb 10;1:786186. doi: 10.3389/frhs.2021.786186. PMID: 36926481;

PMCID: PMC10012749. Accessed 9 September 2023. https://www.ncbi.nlm.nih.gov/pmc/articles/PMC10012749/

Abiiro, G.A., Mbera, G.B. & De Allegri, M. Gaps in universal health coverage in Malawi: A qualitative study in rural communities. BMC Health Serv Res 14, 234 (2014). Accessed 9 September 2023. https://doi.org/10.1186/1472-6963-14-234

RWANDA

Rwanda follows a universal healthcare model, which provides health insurance through the *mutuelles de santé*, a community-based health insurance scheme. Public health insurance is mandatory for all citizens of Rwanda, and in 2010, over 90% of the population was covered. The healthcare system in Rwanda is considered to be one of the highest-quality health systems in Africa, with 499 health centres and 680 health posts. However, the hospitals often struggle to provide basic equipment, electricity, and running water, and many expats prefer to travel to nearby countries for more complicated or delicate medical procedures. The private sector is working hard to improve the specialized medical infrastructure of the country. Rwanda has one medical school located in Butare as part of National University of Rwanda (NUR), and the three referral hospitals serve as the teaching hospitals for the medical school. The actual cost of care per citizen is between USD$14-20, with one half coming from government sources and the remainder from international donors. The private healthcare sector comprises two general hospitals, one eye hospital, 50 clinics and polyclinics, eight dental clinics, four eye clinics, and 134 dispensaries. Private healthcare is growing steadily since 1994, and over 50% of the private healthcare facilities operate near or in the capital of Kigali.

References

Innovations in Healthcare (2020) Universal Health Coverage: How Rwanda is moving forward with healthcare for all. Accessed 1 September 2020. https://www.innovationsinhealthcare.org/universal-health-coverage-how-rwanda-is-moving-forward-with-healthcare-for-all/

Yale (2023) Kigali, Rwanda. Accessed 1 September 2023. https://medicine.yale.edu/intmed/global/sites/rwanda/

Rwanda Development Boarsd, Health Services. Accessed 1 September 2023. https://rdb.rw/investment-opportunities/health-services/

PHILIPPINES

The healthcare system in the Philippines consists of both public and private options.

All Filipino citizens are entitled to free healthcare under the Philippine Health Insurance Corporation (PhilHealth), which is government-organized and funded in part by government subsidies at the local and national level, as well as through company payroll deductions. PhilHealth subsidizes a variety of treatments, including emergency and urgent care, inpatient healthcare, and non-emergency surgeries.

Supplement to: Steele P, Frazer HC, Mekonnen G. People that Deliver theory of change for building human resources for supply chain management: applications in sub-Saharan Africa and Southeast Asia. *Glob Health Sci Pract*. 2025;13(Suppl 1):e2300467. https://doi.org/10.9745/GHSP-D-23-00467

Public hospitals and other public facilities generally handle preventive and primary care in the Philippines. While the overall quality of the Philippines' state-subsidized public healthcare is good, healthcare in rural areas is of significantly lower quality than at hospitals in large cities.

Private facilities provide specialized care in areas such as cardiovascular disease or orthopaedics are are often better equipped than public ones and have the latest equipment and shorter waiting times. Private healthcare is widely available in major cities, and most hospitals in the Philippines are privately run. Someone with diabetes or cancer may only be able to receive necessary care at a private hospital.

While the healthcare system in the Philippines is of a high standard, the infrastructure in most rural medical facilities needs drastic improvements. Expats and those who can afford it often seek care in private settings due to the latest equipment and shorter wait times.

References

International citizens insurance, Healthcare in the Philippines. Accessed 20 September 2023. https://www.internationalinsurance.com/health/systems/philippines.php

Allianz Care, Healthcare in the Philippines. Accessed 20 September 2023. https://www.allianzcare.com/en/support/health-and-wellness/national-healthcare-systems/healthcare-in-philippines.html

Expat financial. Philippines Healthcare System & Medical Insurance Options for Expats. Accessed 19 September 2023. https://expatfinancial.com/healthcare-information-by-region/southeast-asia-healthcare-system-and-insurance-options-for-expats/philippines-healthcare-system-medical-insurance-options-for-expats/
